# Early Therapeutic Plasma Exchange in Pediatric Transverse Myelitis: A Case Report and Scoping Review

**DOI:** 10.3390/neurolint16060122

**Published:** 2024-12-04

**Authors:** Akram Khan, José Peña, Genesis Briceño, Juliann M. Gronquist, Khurram Khan, Raju Reddy, Vijayshree Yadav, Asha Singh

**Affiliations:** 1 Department of Medicine, Division of Pulmonary, Allergy and Critical Care Medicine, Oregon Health and Sciences University, Portland, OR 97239, USA; penaj@ohsu.edu (J.P.); parra@ohsu.edu (G.B.); reddyr@ohsu.edu (R.R.); 2 Department of Nursing, Mirabella Portland, Portland, OR 97239, USA; gronquistj@gmail.com; 3 Department of Anesthesia Saint Luke’s Hospital, Kansas City, KS 64111, USA; khanmd@gmail.com; 4 Department of Neurology, Oregon Health and Sciences University, Portland, OR 97239, USA; yadavv@ohsu.edu (V.Y.); singas@ohsu.edu (A.S.)

**Keywords:** transverse myelitis, therapeutic plasma exchange, corticosteroids, pediatric neurology, case report

## Abstract

Background/Objectives: Transverse myelitis (TM) is a rare, acute inflammatory disorder affecting the spinal cord, with severe potential consequences, particularly in pediatric patients. Therapeutic plasma exchange (TPE) has emerged as a possible intervention for children unresponsive to high-dose corticosteroids. This study explores the efficacy of early TPE in pediatric TM through a case report and scoping review aiming to clarify the therapeutic benefits of TPE when used in conjunction with corticosteroids in children. Methods: We present a scoping review of existing literature on the early administration of TPE in pediatric patients with TM, supplemented by a case report of a 5-year-old boy with Longitudinally Extensive Transverse Myelitis (LETM), who received early TPE and corticosteroid therapy. Clinical progression, response to TPE, and functional outcomes were documented over a 9-month follow-up period. Results: Among the reviewed cases, early TPE demonstrated potential to expedite neurological recovery and improve functional outcomes. In our case report, the patient showed rapid recovery, achieving unassisted ambulation by day four of TPE. No adverse effects were observed. MRI findings revealed substantial resolution of spinal cord lesions by three months, with near-complete symptom resolution at nine months. Conclusions: Early initiation of TPE, in conjunction with corticosteroids, may offer significant therapeutic benefit in pediatric TM, potentially accelerating recovery and improving outcomes. This case highlights the need for further controlled studies to establish evidence-based guidelines for TPE use in pediatric TM.

## 1. Introduction

Transverse myelitis (TM) is a rare inflammatory disorder that leads to acute or subacute motor, sensory, and autonomic (bladder, bowel, and sexual) spinal cord dysfunction [1,2,3,4]. TM can lead to substantial disability, especially in pediatric populations where the disease’s early detection and management pose unique challenges due to symptom overlap with other neurological and inflammatory disorders. Despite the rarity of TM, early recognition and targeted intervention are essential to mitigate potential long-term sequelae. The lack of controlled clinical trials for TM has resulted in no FDA approved therapies specifically targeting this condition. Consequently, treatment decisions are generally based on clinical experience and data from open-label studies and retrospectives analysis, primarily extrapolated from research involving adult populations [1]. Corticosteroids are commonly used as first-line therapy, with recommendations based on case reports or extrapolations from trials in multiple sclerosis (MS) [2,5,6,7,8]. Therapeutic plasma exchange (TPE) is sometimes added to corticosteroid therapy in more severe cases with some reports suggesting it provides additional benefit [9,10,11,12,13]. Other treatments include IV immunoglobulin (IVIG) and cyclophosphamide, although the use of IVIG for transverse myelitis has been “off label” [4,12,14,15,16,17,18].

There is debate in the literature regarding whether the therapeutic protocol for TM should incorporate TPE. Due to the lack of controlled trials in the pediatric population, TPE has been embraced as a rescue therapy [10,11,13,14,15,16,17,19,20,21,22,23,24,25,26,27,28,29,30,31,32]. This case report and scoping review examine the therapeutic role of high-dose corticosteroids combined with therapeutic plasma exchange (TPE) in managing pediatric TM, with a particular focus on refractory cases where corticosteroid response alone is insufficient. Through this report, we aim to evaluate the potential benefits of early TPE initiation in restoring neurological function, reducing hospital stays, and improving outcomes for children with TM.

## 2. Case Report

### 2.1. Patient History and Timeline

**Figure 1:**
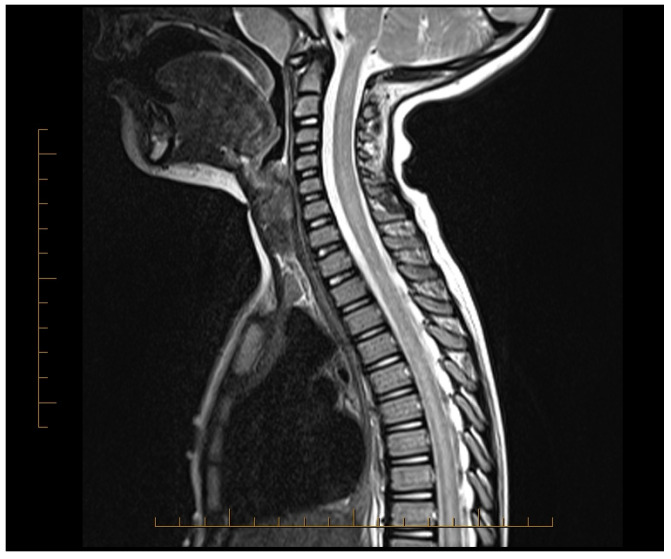
MRI of the spinal cord showing abnormal T2 hyper-intense signal throughout the spinal cord prior to initiating treatment.

A previously healthy 5-year-old boy developed a sore throat, cough, and low-grade fever one week prior to presentation. He was seen by his pediatrician and treated with cefdinir for otitis media. As these symptoms resolved, the patient began experiencing bilateral thigh pain and episodes of priapism, for which he was seen in the emergency room and admitted for observation and urological consultation. The following morning, he developed lower extremity weakness, could only wiggle his toes, and was unable to bear weight on his legs. His lower extremity sensations were reduced bilaterally. Within hours, he developed urinary hesitancy, retention, and constipation. Neurological examination revealed bilateral flaccid paralysis of the lower extremities with areflexia. Magnetic resonance imaging (MRI) of the entire neuroaxis revealed an abnormal T2-weighted hyper-intense signal throughout the cervical, thoracic, and lumbar spinal cord consistent with longitudinal extensive transverse myelitis (LETM), along with a distended bladder and significant stool burden in the distal colon (Figure 1). Lumbar puncture showed normal cerebrospinal fluid (CSF), including a negative aquaporin-4 antibody (NMO antibody) titer, IgG index, oligoclonal bands, and multiple sclerosis panel. He was started on high-dose corticosteroids, 30 mg/kg, within the first 24 h of weakness onset. To address the longitudinally extensive involvement of the spine, concurrent TPE was added to high-dose steroid therapy.

### 2.2. Therapeutic Intervention

Vascular access was established via a double lumen 8-French central venous catheter in the right internal jugular vein. Informed parental consent was obtained, and the patient received his first TPE 20 h after the initiation of corticosteroids. The patient received high-dose corticosteroids (30 mg/kg) for five days followed by an 8-week taper. In addition, he underwent five TPE sessions every other day. Each session involved a 1.5 plasma volume exchange using the COBE Optia device (Terumo BCT, Lakewood, CO, USA) primed with red blood cells (RBCs) for his weight of 18 kg. Albumin was used as replacement fluid with Acid-Citrate-Dextrose Solution-A (ACD-A) as an anticoagulant at a 1:10 ratio. Ionized calcium levels were checked before starting the procedure, midway through the procedure, and at the end. Calcium gluconate (30/mg/kg/h.) was infused and administered during the procedure to prevent hypocalcemia. Baseline PT/PTT and fibrinogen levels were within normal limits, and fibrinogen was checked before each session and was always above 100 mg/dL, and no FFP was used during the procedures.

### 2.3. Follow-Up and Outcomes

The patient tolerated TPE well without any adverse reactions. Within 2–3 h after the first procedure, his family noticed activity in the right thigh, and by the next day, he was able to lift both legs (initially right stronger than the left). After the second TPE session, he was able to walk with a walker, and after the third TPE, the patient was able to walk unassisted. The day after the fourth TPE session, he was able to jump up with no significant change occurred after his final fifth TPE procedure. At the time of discharge (day 12), he was able to ambulate without assistance, with minor leg pain that responded well to ibuprofen and intermittent headaches, attributed to corticosteroids.

**Figure 2:**
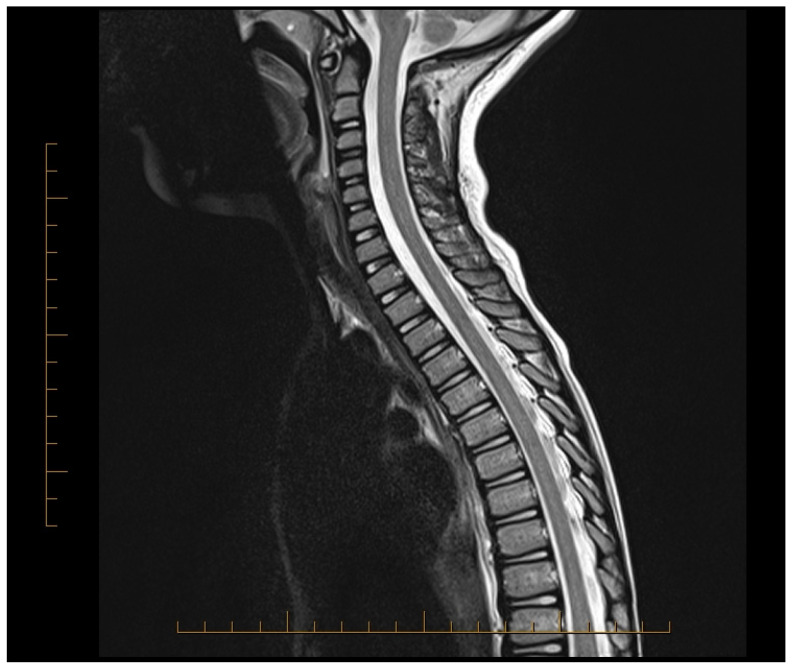
MRI of the spinal cord showing spinal cord lesions improved in appearance after treatment.

Subsequent MRIs of the brain, orbit, and spine revealed normal brain, spine, and orbits (day 12 and 3 months). After 3 months, MRI showed that most spinal lesions returned to normal (Figure 2). Only a minimal cervical lesion remained, which did not enhance with contrast, suggesting an old healing lesion. His brief priapism episodes improved with pelvic physical relaxation therapy after 3 months. Urinary hesitancy also improved with time and relaxation techniques. He returned to his normal motor behavior and was able to swim, bike, and run. At 9 months post-treatment, his only complaint was leg pain, which he rated 1 out of 10 on the pain scale, and mild constipation requiring as-needed polyethylene glycol. His NMO IgG titers remained negative at the onset and at 3 and 9 months. He has been followed clinically for over 4 years with normal function.

## 3. Methods

### 3.1. Literature Search Strategy

This scoping review followed a structured approach to identify relevant studies on the use of TPE in pediatric TM. A literature search was conducted across PubMed, to capture articles published from 2005 to 2024 (20 years). Search terms included combinations of Medical Subject Headings (MeSH) using terms “transverse myelitis,” AND “therapeutic plasma exchange,” using the filters “Case Reports, Clinical Study, Clinical Trial, Clinical Trial, Phase I, Clinical Trial, Phase II, Clinical Trial, Phase III, Clinical Trial, Phase IV, Comparative Study, Evaluation Study, Meta-Analysis, Observational Study, Pragmatic Clinical Trial, Randomized Controlled Trial, English, Humans, Child: birth−18 years, from 2005–2024”.

### 3.2. Inclusion and Exclusion Criteria

Studies were included if they met the following criteria:

1. Reported on pediatric patients (age ≤ 18 years) diagnosed with transverse myelitis or longitudinally extensive transverse myelitis.
2. Documented the use of TPE as a treatment intervention.
3. Provided details on clinical outcomes, including neurological recovery and adverse events.
4. Published in English in peer-reviewed journals.

Studies were excluded if they focused on adult populations, did not involve TPE as a treatment, or lacked adequate outcome data.

### 3.3. Data Extraction, Synthesis, and Quality Assessment

Data extraction was performed independently by two reviewers (A.K., K.K.), who recorded study characteristics, patient demographics, TPE protocols, adjunctive therapies, clinical outcomes, and adverse effects. Discrepancies were resolved by consensus or by consulting a third reviewer (A.S.). Extracted data were synthesized qualitatively to highlight trends, efficacy, and safety of TPE in pediatric TM. The level of evidence and study design were considered when interpreting findings. Case reports, case series, and cohort studies were categorized and examined for potential biases, limitations, and relevance to clinical practice. A meta-analysis or systematic review was not possible due to the heterogeneity of data, and a scoping review was conducted.

## 4. Results

An initial literature search identified 46 articles, all of which were reviewed through a title and abstract review. A total of 23 studies were included in this review, all observational in nature, including case reports, case series, and cohort studies (see Section 5). The available evidence was generally of low quality, with limited or no control groups, small sample sizes (1–90 subjects), and significant variability across studies. The studies spanned diverse geographic locations, each employing unique protocols for TPE administration, including differences in plasma volumes exchanged, frequency of sessions, and adjunctive therapies such as corticosteroids. Due to substantial heterogeneity in study designs, patient populations, treatment protocols, and outcome measures, conducting a meta-analysis was not feasible.

## 5. Discussion

TM is a rare inflammatory disorder of the spinal cord characterized by an acute or subacute onset of motor, sensory, and autonomic dysfunction [1,2,3]. TM has a multifactorial etiology (Table 1) and often emerges as an autoimmune response following an infection or vaccination but can also result from a direct central nervous system (CNS) infection, an underlying autoimmune disease, or as part of a demyelinating disorder such as multiple sclerosis, neuro-myelitis optica spectrum disorder (NMOSD), or acute disseminated encephalomyelitis (ADEM) [2,14,16,17,25,28,30]. Approximately 15–30% of the cases may be idiopathic [2,3,11,33].

In the United States, the annual incidence of acute TM is approximately 1800 cases, with around 20% occurring in children, translating to approximately 300 pediatric cases per year [34]. Pediatric incidence peaks between 0 and 2 years and 5–17 years, with idiopathic myelitis more commonly observed in younger children [32,33]. Additionally, a slight female predominance has been noted in children over 10 years of age, although the disease generally shows no racial predisposition [1,33,35].

Pathologically, TM is marked by focal lymphocytes and monocytes infiltration within the spinal cord, accompanied by varying degrees of demyelination, axonal injury and activation of astroglial and microglial cells [2]. Proposed mechanisms of autoimmune-mediated inflammation in TM include molecular mimicry, superantigen activation, humoral-based dysregulation, and IL-6-mediated toxicity [35].

In a retrospective study by Murphy et al., a comprehensive evaluation of patients initially diagnosed with TM revealed a more specific inflammatory or non-inflammatory etiology in 88% of cases, highlighting the importance of accurate diagnosis for appropriate treatment [33]. Among these patients, idiopathic myelitis constituted 12%, with other cases attributed to specific conditions like MS, NMOSD, and spinal cord infarction. This finding underscores the importance of an etiologic work-up in TM cases to guide tailored clinical management and improve outcomes [33].

The diagnostic criteria for TM, as established by the Transverse Myelitis Consortium Working Group, emphasize bilateral sensorimotor and autonomic dysfunction and a defined sensory level. These criteria, with adjustments for sensory level reporting challenges in young children, are applicable in pediatric cases [1,36].

MRI remains the primary diagnostic tool for assessing and monitoring TM. While extensive documentation exists on MRI findings in adult TM, pediatric imaging data are less robust. Longitudinally extensive TM (LETM) lesions, spanning three or more vertebral segments, are frequently observed in pediatric cases and are especially common in NMOSD and ADEM. Brain MRI abnormalities in children with TM are often predictive of a subsequent MS or NMOSD diagnosis, underlining the importance of brain and spinal imaging in pediatric TM evaluations [1,22]. Although brain lesions are typically absent in idiopathic TM, approximately 40% of children exhibit asymptomatic brain lesions, suggesting potential progression to other demyelinating diseases [1,35,37]. The summary of CRs and studies are shown in Table 2.

The clinical presentation of TM in children can often mimic peripheral nervous system disorders, such as Guillain-Barré syndrome, due to shared symptoms of weakness and areflexia [21,29]. Even after one week of symptom onset, around one-third of TM patients remain undiagnosed [4]. However, the presence of a defined sensory level and symptoms such as urinary retention should suggest spinal cord localization rather than peripheral nerve etiology [36].

The clinical course of transverse myelitis (TM) exhibits considerable variability, with patients typically having no history of neurological abnormalities before disease onset (Table 2) [5]. TM frequently begins after mild illness within the preceding 3 weeks, a finding reported in 50–100% of cases, highlighting a possible post-infectious trigger [1,35]. In pediatric TM, lower back pain is a common presenting symptom. it may involve spinal cord gray matter involvement, reduced activity of modulatory brainstem pathways, and astrocytic damage [5]. Other common symptoms in children include motor deficits, numbness, ataxia, and loss of bladder or bowel control [2,16,19,20]. Less commonly, priapism and visual loss are also observed, reflecting the variability in symptom presentation depending on the spinal cord level affected [9].

Prognosis in pediatric TM varies, with approximately 33–50% of children achieving complete recovery, while 10–20% face significant, lasting impairments (Table 2) [8,12,24,35,37]. Recovery generally begins within weeks of symptom onset, with the most rapid improvements occurring during the first 3–6 months. However, further gains may be observed for up to 2–4 years [9]. In a Canadian study of 38 children with TM, 16% required a wheelchair and 22% experienced ongoing sphincter dysfunction, underlining the potential for substantial residual disability in some patients [35].

Outcomes also differ depending on the underlying etiology of TM. TM associated with acute disseminated encephalomyelitis (ADEM) or multiple sclerosis (MS) tends to have a more favorable prognosis compared to idiopathic TM (ITM) or neuromyelitis optica (NMO)-related TM, which is often associated with more severe residual deficits [2]. Pediatric TM generally carries a better prognosis compared to adult cases; up to 50% of adult TM patients experience poor recovery, whereas approximately two-thirds of pediatric patients achieve a favorable outcome, with only one-third facing persistent disabilities [6].

Due to the lack of controlled clinical trials, there are currently no FDA approved therapies specifically for TM. Treatment decisions are generally based on clinical experience and data from open-label studies and retrospectives analysis, primarily extrapolated from adult studies [1].

Corticosteroids are widely regarded as the first-line therapy for TM, primarily aimed at reducing inflammation and edema in the spinal cord. These recommendations are largely derived from case studies and extrapolated from MS trials, given the similarities in pathophysiology [2]. During the acute phase of TM, the primary therapeutic objective is to halt disease progression and promote resolution of spinal cord inflammation. In post-infectious immune-mediated TM, high-dose corticosteroids serve as the standard initial treatment, with studies in adults suggesting that 50–70% of patients achieve partial or complete recovery and regain ambulation with or without support. However, randomized controlled trials specifically examining corticosteroid use in pediatric TM are lacking [2].

Current guidelines for pediatric TM recommend intravenous methylprednisolone at a dose of 30 mg/kg/day for 5–7 days, followed by a gradual taper of oral steroids over 4–6 weeks [5]. Evidence suggests that early high-dose corticosteroid treatment may shorten the duration of disability and improve long-term outcomes. For instance, a study comparing 12 children treated with high-dose corticosteroids to a historical control group of 17 untreated children found that 66% of the treated group were able to walk within one month, compared to only 17% in the control group [35]. Additionally, 55% of patients in the treated group achieved complete recovery at 12 months, compared to 12% in the control group. The treated group also regained independent walking significantly faster, with an average of 25 days compared to 120 days in the control group. Following intravenous corticosteroids, an oral taper typically begins at 1 mg/kg/day over 3–4 weeks [38]. Potential adverse effects of corticosteroid therapy include gastrointestinal symptoms, insomnia, headache, anxiety, mania, hypertension, hyperglycemia, and electrolyte disturbances.

In cases where patients fail to respond to corticosteroids or experience symptom worsening within 24–48 h, therapeutic plasma exchange (TPE) is recommended as second-line therapy according to American Society for Apheresis (ASFA) category 2 based on clinical experience, case reports, and retrospective analyses [1,5,9,10,11,13,20,39,40,41]. TPE has been used in the treatment of multiple neurological disorders and can be added to standard corticosteroid therapy, particularly in refractory cases (Table 3) [39,40,41]. By removing circulating autoantibodies, cytokines, and immune complexes, TPE aims to mitigate inflammation and potentially accelerate recovery [11,13,39,40,41]. TPE also reduces inflammation via a reduction in complement activation, which decreases complement-mediated inflammation contributing to nerve and tissue injury and reduces pro-inflammatory cytokines (e.g., TNF-α, IL-6), which lowers systemic and local inflammation [39,40,41].

Clinical evidence supporting TPE’s effectiveness in pediatric TM remains limited and heterogeneous, though some studies demonstrate promising results. For example, a retrospective cohort study of 26 children with CNS demyelinating events treated with both TPE and corticosteroids documented clinical improvement in 54% of patients [24]. However, TPE use in TM treatment remains inconsistent across centers. Some institutions reserve TPE for corticosteroid-refractory cases, while others initiate TPE concurrently with steroids in severe presentations [12].

A randomized, double-blind, sham-controlled crossover study involving 22 adult patients with idiopathic acute inflammatory demyelinating syndromes of the CNS (including seven cases of myelitis) who were unresponsive to corticosteroids showed that 42% of the TPE group had moderate improvement compared to only 5.9% in the sham group [42]. Similarly, in a retrospective study of 122 TM patients of various etiologies, 56 who did not respond to corticosteroids received further treatment with TPE, cyclophosphamide, or both. TPE alone was linked to improvement in patients with residual sensorimotor function, though those with complete sensorimotor loss showed better outcomes when treated with both TPE and cyclophosphamide [2]. Additionally, a case series by Magaña et al. highlighted that shorter disease duration and preserved reflexes at the time of TPE initiation were associated with improved responses, suggesting that early TPE may enhance outcomes [43].

A recent study examined outcomes in consecutive pediatric patients with severe CNS demyelination who received TPE within 3.5 weeks of symptom onset [38]. Out of 390 children with confirmed demyelinating disorders, 12 were treated with TPE for an acute demyelinating episode. TPE was initiated between 2 and 24 days post-symptom onset. During hospitalization, 75% of these children showed clinical improvement, with 58% regaining independent ambulation despite significant neurological deficits prior to TPE initiation. However, the contribution of TPE to these outcomes remains inferred, as other factors such as corticosteroid use, remyelination processes, and underlying disease characteristics likely influenced recovery [38].

Despite its potential, TPE carries a slightly higher complication rate in pediatric patients than in adults. Common adverse effects include hypotension, electrolyte imbalances, coagulopathy, thrombocytopenia, and catheter-related issues such as thrombosis and infection [2,10,11,13,39,40,41,44]. In one study, minor side effects were reported in 3 out of 12 pediatric patients treated with TPE, though these were generally manageable [38]. A study of 186 children who had undergone 1632 apheresis procedures (including TPE, hematopoietic progenitor cell collection, red cell exchange, and leukodepletion) found that 55% of procedures were associated with adverse effects [45]. Most of the adverse reactions were benign and related to citrate toxicity and volume shifts. Another pediatric cohort evaluating TPE for CNS acute events like TM and optic neuritis reported 72% neurological improvement at the three-month follow-up, with a manageable 5.9% incidence of mild-to-moderate adverse events, which were promptly treated or resolved spontaneously [13].

Due to the need for specialized personnel and equipment, TPE may not be readily accessible in all treatment centers, particularly in community settings. Nonetheless, many clinicians consider TPE more effective than IVIG therapy in managing severe TM cases. The American Academy of Neurology guidelines recommend that clinicians consider TPE in addition to corticosteroids for patients with TM when first-line treatments are insufficient [4].

Intravenous immunoglobulin IVIG and cyclophosphamide have been used in both pediatric and adult TM cases. IVIG, while FDA-approved for various autoimmune disorders, is considered “off label” for TM [4,17,30,46]. Cyclophosphamide, typically administered at a dose of 500–750 mg/m^2^, has also been considered in refractory cases [35]. However, data on IVIG and cyclophosphamide in TM are limited, and these therapies are typically reserved for cases where first-line treatments, like corticosteroids or TPE, are ineffective. They will not be discussed further in this review focused on the role of TPE.

## 6. Conclusions

Transverse myelitis (TM) represents a rare but severe inflammatory condition with potentially debilitating outcomes, particularly in pediatric patients. However, early and appropriate management holds significant potential for improving outcomes. The literature, alongside this case report documenting successful concurrent treatment with high-dose corticosteroids and therapeutic plasma exchange (TPE) in a pediatric patient, supports the role of early TPE initiation in facilitating rapid neurological recovery, reducing hospital stays, and enhancing overall prognosis in TM. While TPE is associated with a higher incidence of adverse reactions in children compared to adults, these reactions are generally mild and transient, reinforcing TPE’s favorable safety profile in the pediatric population. Given the promising results observed, further prospective studies are warranted to systematically evaluate the efficacy, optimal timing, and safety of TPE in pediatric TM, potentially establishing it as a standard adjunctive treatment in severe or corticosteroid-resistant cases.

## Figures and Tables

**Table 1:** Etiological Classification and Specific Causes of Transverse Myelitis. This table categorizes the etiologies of transverse myelitis (TM) based on associated conditions and potential causative factors. The table highlights common presentations and characteristics for each category, offering insights into TM’s diverse and often complex underlying mechanisms. Abbreviations: ADEM: Acute Disseminated Encephalomyelitis, AFM: Acute Flaccid Myelitis, CNS: Central Nervous System, CRMP5: Collapsin Response Mediator Protein 5, HIV: Human Immunodeficiency Virus, HTLV-1: Human T-Lymphotropic Virus Type 1, MOGAD: Myelin Oligodendrocyte Glycoprotein Antibody-Associated Disease, MS: Multiple Sclerosis, NMOSD: Neuromyelitis Optica Spectrum Disorder, SCLC: Small Cell Lung Cancer, SLE: Systemic Lupus Erythematosus, TM: Transverse Myelitis.

Etiology Category	Specific Causes	Notes
CNS Demyelinating Disorders	Multiple sclerosis (MS) Neuromyelitis optica spectrum disorder (NMOSD) Myelin oligodendrocyte glycoprotein antibody-associated disease (MOGAD) Acute disseminated encephalomyelitis (ADEM)	TM can be an initial event in MS or appear as a feature in NMOSD, MOGAD, and ADEM, each with characteristic manifestations (e.g., longitudinally extensive lesions in NMOSD, and monophasic symptoms in ADEM).
Systemic Autoimmune Disorders	Sarcoidosis Sjögren’s syndrome Systemic lupus erythematosus (SLE)	TM may result from these systemic inflammatory diseases, with varying mechanisms of central nervous system involvement.
Less Common Systemic Autoimmune Disorders	Ankylosing spondylitis Antiphospholipid syndrome Behçet syndrome Mixed connective tissue disease Rheumatoid arthritis Systemic sclerosis	These conditions are less frequently associated with TM but can present neurologic involvement affecting the spinal cord.
Infections	Enteroviruses (e.g., D68, EV71) West Nile virus Herpes viruses HIV HTLV-1 Zika virus Neuroborreliosis (Lyme disease) Mycoplasma Treponema pallidum	Infectious agents are rare causes of TM; enteroviruses are linked to acute flaccid myelitis (AFM), which mimics polio-like symptoms and occurs in outbreaks.
Paraneoplastic Syndromes	Associated with antibodies: anti-Hu, anti-CRMP5, anti-amphiphysin Commonly associated malignancy: Small cell lung cancer (SCLC)	TM related to paraneoplastic syndromes may present with other nervous system symptoms and is often linked to specific autoantibodies associated with malignancies like SCLC.
Vaccinations	Reported temporal association with vaccines	TM has been temporally associated with vaccines, but causation is unproven. Studies have found no significant correlation between TM and recent vaccination.
Idiopathic	No identifiable cause in 15–30% of cases	Idiopathic TM is diagnosed when no specific cause is found despite thorough evaluation. Many cases report an antecedent illness (respiratory, GI, or systemic) but lack definitive evidence of causation.

**Table 2:** Summary of CRs and Studies on Transverse Myelitis and Related Disorders: Clinical Characteristics, Interventions, and Outcomes. Data from 23 studies of TPE use and 6 studies without TPE use. Abbreviations: ADEM: Acute Disseminated Encephalomyelitis, AFM: Acute Flaccid Myelitis, AQP4-IgG: Aquaporin-4 Immunoglobulin G, APS: Antiphospholipid Syndrome, CNS: Central Nervous System, CMP: Cyclophosphamide, CR: Case report, CS: Case series, CSF: Cerebrospinal Fluid, EDSS: Expanded Disability Status Scale, F: Female; GBS: Guillain-Barré Syndrome, HSV: Herpes Simplex Virus; IVIG: Intravenous Immunoglobulin, LETM: Longitudinally Extensive Transverse Myelitis, MOG: Myelin Oligodendrocyte Glycoprotein, M: Male; MP: Methylprednisolone, MRC: Medical Research Council, MRI: Magnetic Resonance Imaging, MS: Multiple Sclerosis, NMOSD: Neuromyelitis Optica Spectrum Disorder, RCT: Randomized Controlled Trial, TM: Transverse Myelitis, TPE: Therapeutic Plasma Exchange, WeeFIM: Functional Independence Measure for Children.

Year	First Author	Type of Study	Location	No. of Subjects	Age (Years)	Sex	Case History	Treatment	Outcome
2006	Arabshahi [17]	CR	Philadelphia, Pennsylvania USA	1	11	F	NMOSD as initial presentation of primary Sjögren’s syndrome with optic neuritis and TM	MP CMP TPE	Remission with treatment led to remission, suggests potential overlap between NMOSD and other autoimmune conditions
2007	Pidcock [32]	CS	Baltimore, Maryland, USA	47	0–17	24 M 23 F	Acute TM, median follow-up 3.2 years. Evaluated functional outcomes and prognostic factors affecting recovery.	Variable	Long-term, 43% of children were unable to walk 30 feet, 68% experienced urinary urgency, and 75% had ongoing numbness. Age < 3 years associated with worse functional outcomes.
2009	Csábi [31]	CR	Pécs, Hungary	1	14	M	TM associated with mycoplasma pneumoniae presented with paraplegia + bowel/bladder dysfunction	MP TPE Roxithromycin	No recovery for patient, potential for persistent disability in mycoplasma-associated TM
2011	Rodrigues [30]	CS	São Paulo, Brazil	14	8 to 83	F	TM associated with APS, majority of pts experiencing severe symptoms like sphincter dysfunction and thoracic spinal cord involvement	MP CMP TPE	64% had complete recovery
2014	Meyer [37]	CS	Montpellier, France	30	3–15 (median 11)	14 M 16 F	Not specified	87% MP 50% IVIG 37% both	60% probability of relapse at 5 years, 80% of patients able to walk independently within 1 month, Children with acute TM and brain MRI abnormalities may later develop MS.
2014	DeSena [29]	CS	Dallas Texas, USA	5	1–13 2–14 3–9 4–8 5–29	1-F 2-M 3-F 4-M 5-F	TM and ADEM pts with atypical peripheral nerve involvement-leading to worse prognoses than typical TM/ADEM cases	MP TPE	1. Unable to ambulate with unilateral support; urinary retention resolved. 2. Urinary retention improved; urgency and occasional incontinence persist. 3. Fully recovered at 3-month follow-up. 4. Unable to walk; requires bilateral support to stand. 5. Deteriorated post-steroid taper; new brain MRI lesions required re-escalation of steroids
2014	Sarioglu [28]	CR	Izmir, Turkey	1	25 months	M	Developed TM after HSV infection presented with urinary retention, respiratory distress, and progressive weakness; neuroimaging showed CNS lesions consistent with ADEM	MP TPE	Significant residual motor impairment
2016	Suthar [6]	CS	North India	36	All patients < 12 years old. Median age: 7.5 years (Only children under 12 are admitted to this hospital)	21 M 15 F	Symmetrical weakness at onset, progressing over 2 days. Thoracic cord most often affected. Median follow-up 35 months: 15 non-ambulatory or needed assistance.	MP (all). 3 children received acyclovir 4 children received azithromycin.	Worse outcomes linked to severe weakness (MRC ≤ 1), spinal shock, respiratory muscle weakness, mechanical ventilation, or delayed diagnosis/treatment.
2017	Hsu [27]	CR	Taipei, Taiwan	1	12	M	Sudden onset low back pain followed by quadriplegia, hyperalgesia, flaccid bladder, and altered consciousness. MRI showed diffuse T2 hyperintensity from cervical cord to conus medullaris.	MP TPE	After TPE, limb function improved, bladder/rectal issues resolved. At 6 months: independent eating, supported ambulation, EDSS 5
2017	Absoud [9]	RCT	United Kingdom	2	Child aged 10–15 years, adult aged 60–65	F child Adult M	Despite recruiting (26 across 15 centers in the U.K.) only two patients randomized-- due to strict inclusion criteria.	MP IVIG	Impact of the use of IVIG in addition to standard therapy children/adults with TM/NMO could not be determined due to low enrollment.
2017	Fukuoka [25]	CR	Osaka, Japan	1	3	F	TM presented with limb weakness, urinary retention, and respiratory issues	MP IVIG TPE	Rehabilitation improved motor function--but became bedridden and upper limb-predominant paralysis remained 2 years after onset, experienced two cardiac arrests requiring pacemaker
2017	Fukuoka [26]	CR	Osaka, Japan	1	11	M	Idiopathic TM: developed progressive hypesthesis, urinary dysfunction, and significant motor impairment	MP IVIG TPE	Hemorrhagic spinal lesions, complete recovery after 4 months
2018	Noland [12]	CS	Dallas, Texas	19	7 months –17	11 F 8 M	Review of patients receiving TPE for TM.	TPE	79% had major improvements in symptoms after 4–7 TPE Majority required further inpatient or outpatient physical therapy 4 patients returned to baseline, 75% regained ability to ambulate 4 adverse events noted over 114 treatments
2019	Chawla [7]	CR	Delhi, India	1	5	Not reported	Presented with fever, rapid paraparesis, upper limb weakness, urinary retention, and constipation. Had hypertonic lower limbs, extensor plantar reflexes, and absent abdominal reflexes MRI had signal abnormalities in the spinal cord	MP	Gradual improvement of power, complete recovery by 5th week.
2019	Manguinao [24]	CS	San Francisco, California, USA	26	2–17	16 F 10 M	Acute events: MS (*n* = 15), NMOSD (*n* = 7), MOG (*n* = 2), TM (*n* = 1), ADEM (*n* = 1). Diagnoses included MS, NMOSD, TM, ADEM, or MOG-antibody demyelination with disease onset and TPE treatment before age 18	MP TPE	14/24 patients improved after PS; 13 improved further with TPE, and 8/10 non-responders to PS improved with TPE. Median EDSS: pre-TPE 4.0, post-TPE 3.75. Adverse events included hypotension, thrombocytopenia, spinal hemorrhage, and GI discomfort.
2019	Savransky [13]	CS	Buenos Aires, Argentina	65	Median age at TPE: 10.5 (1.9–18)	36 M 29 F	Mixed Optic Neuritis and LETM, 20/42 pts had seropositivity immunoglobin G/myelin oligo. glycoprotein	TPE	72% showed neurologic improvement; at 6 months, 88.5% improved. Benefits included lower EDSS scores, better visual outcomes, and gait scales. Adverse events occurred in 5.9% of procedures, with 4 severe cases out of 524, TPE effectively rescued severe steroid-unresponsive cases, improving function with low adverse event frequency
2019	Thabah [23]	CR	India	1	18	F	Presented with LETM and diagnosed with NMOSD (indicated by high AQP4-IgG antibody titers)	MP TPE maintenance with prednisolone, azathioprine, and hydroxychloroquine	pt responded well to initial treatment and TPE, relapse free at 1 year
2020	Ashfaq [8]	CS	Pakistan	34	1–13	20 M 14 F	73.5% with acute paraplegia, 26.47% with quadriplegia; 71% had prior febrile illness	MP	41.2% achieved complete recovery, 58.8% partial recovery, with outcomes linked to gender, spinal cord involvement, muscle power, and autonomic dysfunction.
2020	Ganelin-Cohen [22]	CS	Israel	23	0.5–17	11 M 12 F	Pediatric TM patients (<18 years) with non-recurrent demyelinating events presented with limb paresis, paresthesia, sensory disturbance, sphincter dysfunction, and back pain. CSF showed pleocytosis; mean follow-up: 6 years, 10 months	Variable	Factors associated with prognosis: CSF pleocytosis, no quadriparesis, prolonged time to nadir; 70% good outcome, 30% poor
2021	Celik [5]	CS	Turkey	15	1–15	7 M 8 F	Symptoms: inability to walk (12), incontinence (9), back pain (4), abdominal pain (2), arm weakness (2). Barthel index: 8 independent, 3 moderately dependent, 2 slightly dependent.	MP	1-month: improved muscle strength. 6-month: 2 with stable monoparesis, 2 with paraparesis. Paraparesis reduced from 80% to 13.3%. 66.6% fully recovered during follow-up, results support MP and early TPE if steroids ineffective
2021	PCORI Greenberg [4]	CS	Washington, DC, USA	90	0–18	54 M 36 F	Children diagnosed with TM or AFM within 6 months; MS and NMOSD excluded.	MP IVIG TPE	TPE First-Line: 3/3 (100%) achieved ≥ 22-point WeeFIM increase; no significant risk difference vs. non-TPE (*p* = 0.24). IVIG First-Line: 4/8 (50%) achieved ≥ 22-point WeeFIM increase.
2021	Khera [21]	CR	Jodhpur, India	1	11	F	Developed LETM and GBS post-COVID-19 with febrile illness and severe paralysis needing ventilatory support	MP TPE	improved after 2 weeks of hospitalization, extubated, able to walk at 6 weeks
2022	Poyrazoğlu [15]	CR	Elazığ, Turkey	1	10	M	Presented with ADEM and TM after COVID-19 infection: severe weakness and inability to walk	MP IVIG TPE	patient regained partial function after treatments
2023	Fjellbirkeland [11]	CR	CopenhagenDenmark	1	16	F	16 y F with ADEM: 2-week history of headaches, nausea, neck pain. CSF: monolytic pleocytosis, low CSF glucose, elevated protein, increased lactate	MP TPE	1 month: normal motor function, fatigue, headaches. 3 months: back to school, intermittent pain, incontinence resolved in a year.
2023	Yoo [16]	CR	Seoul, Korea	1	10	F	COVID-19 positive with rapid motor and sensory deficits: neck pain, leg weakness, urinary dysfunction	MP IVIG TPE	6 months: motor function returned, significant neurological deficits remained
2023	Akçay [14]	CR	Istanbul, Turkey	1	9	F	TM following COVID-19 with severe symptoms and rapid worsening	MP IVIG TPE	TPE significantly improved neurological symptoms
2024	Aljezani [10]	CS	Jeddah, Saudi Arabia	15	2–18	12 M 3 F	2 patients with TM	MP TPE	TPE improved outcomes
2024	Tapia-Fonseca [20]	CR	Monterrey, Mexico	1	15	M	Presented with cervical-dorsal medullary syndrome: ascending symmetric paraparesis rapidly progressing to paraplegia (T3 sensory level). COVID-19 positive.	MP TPE	Slow recovery following treatment
2024	Lafian [19]	CR	California, USA	1	18	F	Presented with intractable hiccups and paraparesis--MRI showing T2 hyperintensity from the medulla to the conus	MP TPE Rituximab	Complete recovery, serology for west nile virus positive

**Table 3:** Pathophysiology of Neurological Disorders and Role of TPE.

Neurological Disorder	Pathophysiology	Role of TPE	Clinical Use
Myasthenia Gravis (MG)	Autoantibodies target acetylcholine receptors, impairing neuromuscular transmission.	Removes autoantibodies, improving neuromuscular function, especially during crises.	Acute exacerbations or preoperative stabilization.
Guillain-Barre Syndrome (GBS)	Immune-mediated attack on peripheral nerve myelin or axons, often triggered by infection.	Clears anti-ganglioside antibodies, halting progression and accelerating recovery.	Most effective within two weeks of onset, reduces ventilatory dependence.
Chronic Inflammatory Demyelinating Polyneuropathy (CIDP)	Chronic immune-mediated demyelination of peripheral nerves.	Reduces immune factors causing nerve damage and improves function.	Long-term management alongside immunosuppressants.
Neuromyelitis Optica Spectrum Disorders (NMOSD)	Autoantibodies (e.g., anti-aquaporin-4) target astrocytes in the CNS, causing severe demyelination.	Removes anti-aquaporin-4 antibodies, mitigating acute relapses.	Acute relapses, combined with corticosteroids.
Autoimmune Encephalitis	Autoantibodies target neuronal or synaptic proteins, leading to neuroinflammation.	Clears pathogenic antibodies, reducing inflammation and preventing long-term damage.	During acute episodes, in conjunction with immunotherapy.
Multiple Sclerosis (MS)	Autoimmune-mediated demyelination of CNS neurons, causing acute relapses.	Removes autoantibodies exacerbating demyelination, effective in steroid-refractory relapses.	Second-line treatment for severe relapses not responding to steroids.

## Data Availability

Data are available on request.
